# Unveiling the Chemical Diversity of the Deep-Sea Sponge *Characella pachastrelloides*

**DOI:** 10.3390/md20010052

**Published:** 2022-01-05

**Authors:** Sam Afoullouss, Anthony R. Sanchez, Laurence K. Jennings, Younghoon Kee, A. Louise Allcock, Olivier P. Thomas

**Affiliations:** 1 Marine Biodiscovery, School of Chemistry and Ryan Institute, National University of Ireland Galway (NUI Galway), University Road, H91TK33 Galway, Ireland; S.AFOULLOUSS1@nuigalway.ie (S.A.); LAURENCE.JENNINGS@nuigalway.ie (L.K.J.); 2 School of Natural Sciences and Ryan Institute, National University of Ireland Galway (NUI Galway), University Road, H91TK33 Galway, Ireland; louise.allcock@nuigalway.ie; 3 Department of Cell Biology, Microbiology, and Molecular Biology, University of South Florida, 4202 E. Fowler Ave., Tampa, FL 33620, USA; anthony.sanchez@austin.utexas.edu (A.R.S.); ykee@dgist.ac.kr (Y.K.)

**Keywords:** deep-sea, sponge, cyanocobalamin, molecular networking, poecillastrins, hercynine, *Characella*

## Abstract

Sponges are at the forefront of marine natural product research. In the deep sea, extreme conditions have driven secondary metabolite pathway evolution such that we might expect deep-sea sponges to yield a broad range of unique natural products. Here, we investigate the chemodiversity of a deep-sea tetractinellid sponge, *Characella pachastrelloides*, collected from ~800 m depth in Irish waters. First, we analyzed the MS/MS data obtained from fractions of this sponge on the GNPS public online platform to guide our exploration of its chemodiversity. Novel glycolipopeptides named characellides were previously isolated from the sponge and herein cyanocobalamin, a manufactured form of vitamin B_12_, not previously found in nature, was isolated in a large amount. We also identified several poecillastrins from the molecular network, a class of polyketide known to exhibit cytotoxicity. Light sensitivity prevented the isolation and characterization of these polyketides, but their presence was confirmed by characteristic NMR and MS signals. Finally, we isolated the new betaine 6-methylhercynine, which contains a unique methylation at C-2 of the imidazole ring. This compound showed potent cytotoxicity towards against HeLa (cervical cancer) cells.

## 1. Introduction

Deep-sea habitats at 200 m–2000 m on the continental margin may be highly diverse, especially in submarine canyon systems, where hydrography concentrates food resources [1]. Such habitats are rich in sponges and corals [2], taxa whose holobionts represent the most promising sources of bioactivity [3]. Sponges alone have yielded nearly 50% of the marine natural products between 1990 and 2009 [4], only recently being surpassed by microbes [5]. Several factors promote the evolution of novel chemical architectures in deep-sea sponges: sessile organisms may evolve secondary metabolites that act as chemical defenses against biofouling and predation [6], while extreme environmental conditions can drive adaptations in biochemical pathways [3,7]. However, there are known difficulties in collecting deep-sea samples, and of the ~9620 compounds isolated from sponges, only ~290 were isolated from specimens collected from below 200 m [8].

In our quest for bioactive compounds from Irish deep-sea sponges, we focused on a tetractinellid sponge *Characella pachastrelloides*, collected in Whittard Canyon, one of the largest submarine canyon systems in the NE Atlantic. We previously identified and described four unique glycolipopeptides named characellides with anti-inflammatory properties from this sponge [9,10]. The order Tetractinellida is known for its rich diversity of bioactive compounds. To date, the natural product chemistry of few deep-water tetractinellids has been investigated, but those few have yielded a variety of natural products including bisindole alkaloids [11], cytotoxic peptide lactones [12], macrolides [13,14] and other polyketides [15], steroidal saponins [16], and cytotoxic linear acetylenes [17], suggesting this group has potential for yielding new bioactive metabolites.

Where species are chemodiverse, as seen in tetractinellids, molecular networking can aid in distinguishing and identifying the different families of natural products present. Molecular networking compares MS/MS spectra of metabolites with known or calculated MS/MS spectra and provides a visual representation of compound similarity, clustering structurally similar natural products together [18]. Molecular networking can aid in dereplication but can also help identifying multiple closely related compounds and analogs occurring in a complex mixture, allowing the components of extracts and fractions to be more easily discerned. We applied molecular networking to our MS/MS data and revealed clusters of diverse families of natural products that we explored in depth. Herein, we report on the presence of characellide analogs **1**–**4**, polyketide poecillastrins **5** and **6**, cyanocobalamin **7**, and a new histidine derivative, 6-methylhercynine (**8**).

## 2. Results and Discussion

### 2.1. Molecular Networking

**Figure 1:**
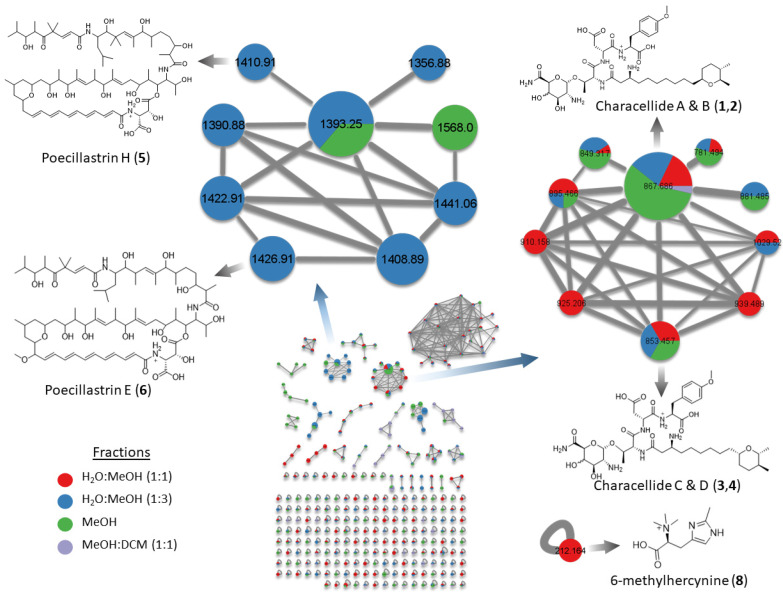
Molecular network of *Characella pachastrelloides* fractions, with annotated metabolites. Pie charts indicate metabolites distribution in fractions (sum precursor ion intensity). Size of node is relative to precursor ion intensity. Edge width increases with higher cosine score.

In an attempt to inspect the chemical diversity of the deep-sea sponge *Characella pachastrelloides*, we first built molecular networks through the online Global Natural Products Social (GNPS) platform [18]. The extract prepared from *C. pachastrelloides* using a mixture of solvents CH_2_Cl_2_/MeOH (1:1) was fractionated using C18 Solid Phase Extractions (SPE) into five fractions of decreasing polarity from H_2_O to MeOH and then CH_2_Cl_2_. All, but the first aqueous fraction, were analyzed by UHPLC-HRMS/MS on a QToF instrument. MS/MS data from each analyzed fraction were processed and combined to produce one molecular network for the chemical diversity of the deep-sea sponge *Characella pachastrelloides*. Molecular networks were generated using both the CLassical Molecular Networking (CLMN) and the Feature Based Molecular Networking (FBMN) workflows. CLMN showed the presence of three large clusters (>7 nodes) and eighteen small clusters (2–7 nodes). Twenty-nine nodes were hit against GNPS libraries including fatty acid derivatives, and dipeptides. FBMN was preprocessed using MZmine2 and produced a network with three large clusters and seven small clusters, with only six nodes hitting against GNPS library spectra. These hits include the same fatty acid derivatives annotated in the CLMN. Dereplication was carried out manually, comparing predicted molecular formulae against marine natural product libraries such as MarinLit. Manual annotation was added by node MS2 peaks visualizer tool available on the online network analyzer, to find nodes sharing similar fragment. The CLMN was visualized using Cytoscape (Figure 1).

### 2.2. Glycolipopeptides: Characellides

A first cluster contained 10 nodes with molecular masses between *m*/*z* ~853 and 1029 including the four previously described novel glycolipopeptides, characellide A–D (**1**–**4**) [9]. Further analysis of this cluster in FBMN revealed the presence of two nodes, 854 and 868, absent in the CLMN. Interrogation of MS/MS spectra of both nodes suggested there were analogs of characellides A and C, replacing a sugar unit containing two nitrogen *m*/*z* 175.07 (A_α_) for sugar unit containing one nitrogen *m*/*z* 176.06 (A_α_) (Figure S1). Characellide B has since been synthesized [19], showing that the configurations of this compound differ from those of the proposed structure of the natural product. Four isomers synthesized with various _L_ and _D_ peptides were not in agreement with NMR data of the isolated natural product, leaving the configuration between the peptidic chain and the sugar unit the most likely source of the differences observed. An in-depth analysis of the MS/MS spectra between annotated and unannotated nodes allowed us to hypothesize the structure of other characellides C and D (3–4) in the cluster. Isolation of the new characellides was attempted but due to low quantities and the difficulty of sample collections, these attempts were unsuccessful.

### 2.3. Polyketides: Poecillastrins

A second cluster of the molecular network only detected in CLMN contained analogues of chondropsin macrolide lactams such as poecillastrin H (**5**) that was recently isolated from a deep-sea sponge, *Characella* sp. [20]. From this annotated node, we also identified the presence of poecillastrin E (**6**), which has been isolated from a deep-sea tetractinellid sponge [13]. The presence of poecillastrin H was confirmed by characteristic **^1^**H-NMR signals at δ_H_ 7.23 and doublet at δ_H_ 6.94 matching those found in the literature. Additional characteristic UV absorption at 370 nm was observed. Chondropsins are known to have potent cytotoxicity caused by selective inhibition of V-ATPases [21]. The presence of unidentified nodes, with a high ratio of edges per node, within this cluster suggested the presence of undiscovered analogs. Retrieving the nodes’ retention times and molecular weights from the molecular networking allowed us to carry out a targeted purification of these macrolides. Due to their extreme photosensitivity, the chemical structure of theses analogs could not be determined. These compounds may be only stable in the absence of light as in their natural environment.

### 2.4. Cyanocobalamin

**Figure 2:**
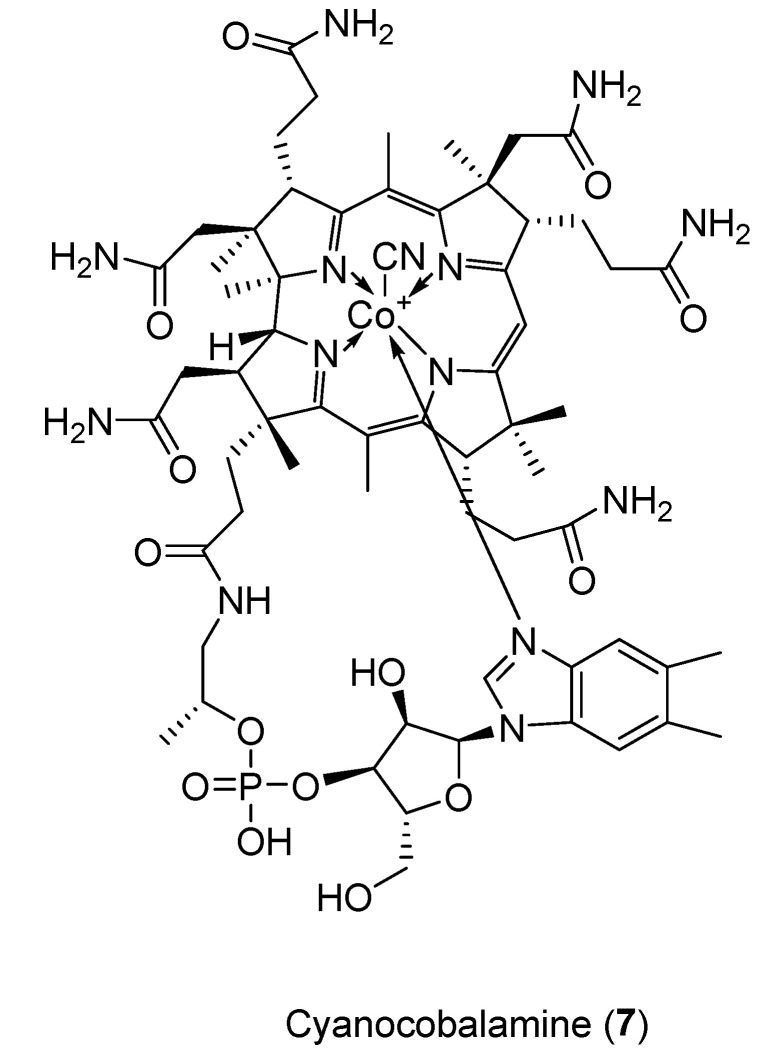
Structure of cyanocobalamin (**7**).

Compound **7** was isolated (4.4 mg) as a pink amorphous solid. A molecular formula of C_63_H_88_CoN_14_O_14_P was revealed using HRESIMS with *m*/*z* 1355.5719 [M + H]^+^ (Figure 2). Cyanocobalamin was identified in the literature with this exact molecular formula (CN-B_12_). This was confirmed by comparing HSQC spectra of compound **7** and a cyanocobalamin standard by NMR. This compound was not observed in the molecular networks.

Cobalamin or vitamin B_12_ derivatives are members of the corrinoids, characterized by their cobalt-coordinated corrin ring. There are multiple vitamers of B_12_, with axial ligands varying from hydroxyl, methyl, cyano or 5′-deoxyadenosyl, with cyanocobalamin being the most stable. Vitamin B_12_ plays an intrinsic role in metabolic processes as a co-enzyme, making it vitally important in the medical field. It is produced by bacteria and archaea and its biosynthesis is well studied [22]. From a review of the literature, this appears to be the first time cyanocobalamin has been isolated from a natural source. Mass spectrometry analysis of the extract was carried out to determine whether any other forms of Vitamin B_12_ were present, with cobalamine found to be present in minute amounts. As it is possible for cyanocobalamin to be produced as a byproduct from others forms of B_12_, due to the affinity of those forms for cyanide, the absence of other vitamers indicated that the cyanocobalamin was not an artefact. This leaves two possibilities for the origins of the cyanocobalamin. First, it could be produced by prokaryotes living within the sponge, potentially via a new biosynthetic pathway. This may be a result of the extreme environmental conditions of the deep sea (pressure, temperature etc.) encouraging the production of the more stable cyanocobalamin over the usually produced hydroxocobalamin. Since biosynthesis of Vitamin B_12_ is limited to bacteria and archaea, the presence of cyanocobalamin indicates that deep-sea sponges, just like their shallow-water counterparts, host a productive microbial community. Second, cyanocobalamin could be accumulated within the sponge, aided by its filter feeding nature. Interestingly, in the marine world, the major producer of Vitamin B_12_ is Thaumarchaeota [23].

### 2.5. Betaine: 6-Methylhercynine

**Figure 3:**
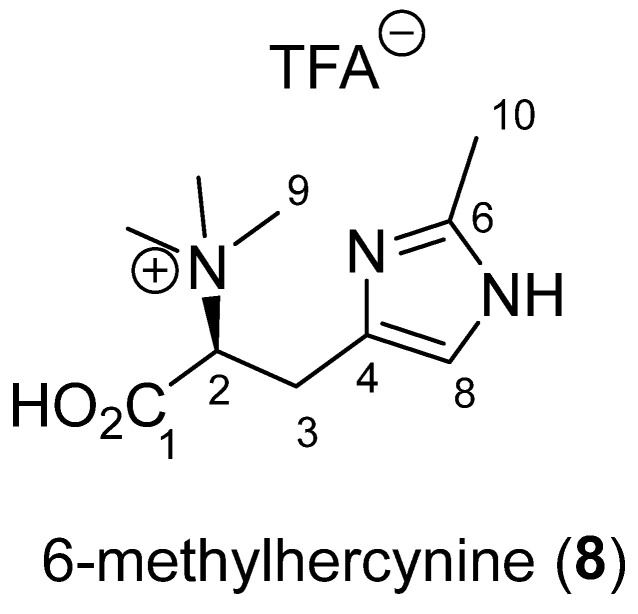
Structure of the new histidine derivative 6-methylhercynine (**8**).

Compound **8** was isolated as a white amorphous powder. Its molecular formula was determined by HRESIMS to be C_10_H_17_N_3_O_2_ with *m*/*z* 212.1394 for [M + H]^+^ (Figure 3). ^1^H-NMR data revealed the presence of four methyl singlets at *δ_H_* 3.30 (s, 9H, H-9), 2.71 (s, 3H, H-10), one aromatic methine at *δ_H_* 7.27 (s, 1H, H-8), and an ABX system at *δ*_H_ 3.97 (dd, 1H, *J* = 12.0, 3.5 Hz, H-2), 3.44 (dd, 1H, *J* = 14.0, 3.5 Hz, H-3a) and 3.37 (dd, 1H, *J* = 14.0, 12.0 Hz, H-3b) (Table 1). HSQC and HMBC spectra of **8** indicated resonances associated with three non-protonated carbons: a carboxylic acid at *δ_C_* 170.2 (C-1) and two aromatic carbons at *δ_C_* 144.6 (C-6) and 128.3 (C-4). The only spin coupled system (SCS) was assigned through H-2/H-3a and H-3b COSY correlations. The H-3/C-1 and H-9/C-2 HMBC correlations established the presence of an *N*-trimethylated amino acid derivative. The connection between the aromatic ring and the SCS was evidenced by key H-3a and 3b/C-4 and C-8 HMBC correlations. The chemical shifts of the aromatic signals at C-4, C-6 and C-8 suggested the presence of an imidazole ring but one aromatic proton was missing in the ^1^H NMR spectrum. The fourth methyl at C-10 was located on the aromatic ring at C-6 due to a H-10/C-6 HMBC correlation. H-10 had only one correlation in HMBC confirming the presence of a substituted imidazole ring.

**Figure 4:**
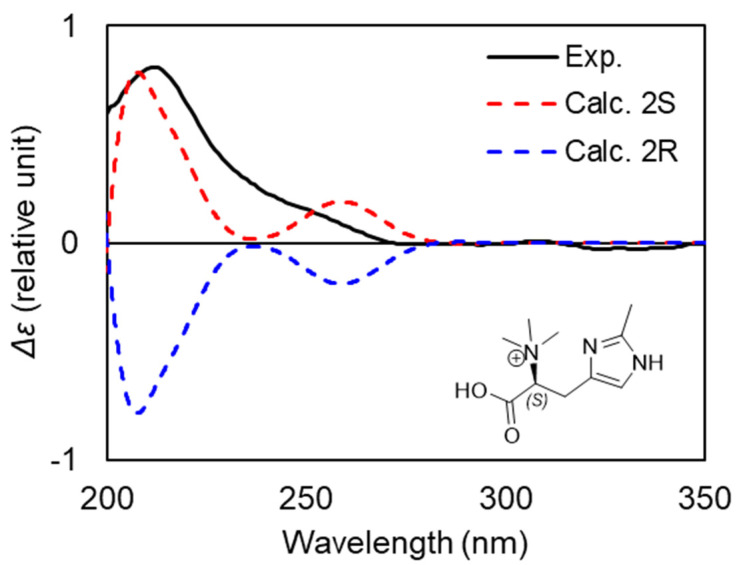
Comparison between the experimental and calculated ECD spectra for two enantiomers of compound **8**.

The absolute configuration at C-2 was determined by comparison between experimental and calculated electronic circular dichroism (ECD) spectra (Figure 4). After a conformational analysis and geometry optimization, the ECD spectra of the two possible enantiomers of **8** were calculated using time-dependent density functional theory (TDDFT). The electronic transition and rotational strength calculations were conducted at the B3LYP/6-11G(d,p) level with 50 transition states. The charge state of the molecule was predicted to be +1 due to the use of acidic conditions during RP-HPLC purification. However, the neutral zwitterion and doubly charged ion were also calculated for comparison with experimental data. Spectra were Boltzmann weighted based on a free-energy distribution and corrected with the UV data. In the calculated spectra of the (2*S*) enantiomer for all charged states, a positive Cotton effect at 215 nm that matches the experimental spectra of **8** was observed, allowing the assignment of (*S*)-6-methylhercynine. Moreover, the experimental spectra closely match previously published experimental spectra of L-histidine and *S*-hercynine, further supporting our TDDFT assignment [24,25].

**Scheme 1:**
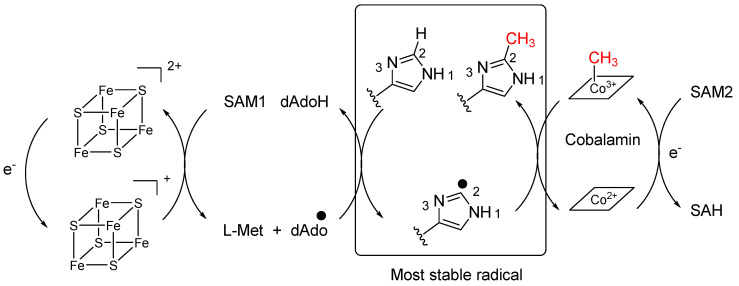
Proposed metabolic pathway for **8**.

This new betaine is a methylated derivative at C-6 of hercynine and, to the best of our knowledge, it is the first natural product where an imidazole is methylated at this position. Indeed, positions *N*-1 and *N*-3 are the most reactive nucleophilic centers for an imidazole and position C-2 is rarely substituted. Searching in the literature, we could only identify synthetic compounds with a methyl at C-2 of an imidazole ring. They were obtained by chemical synthesis using radical conditions with silver salts [26,27]. Hercynine, the betaine corresponding to histidine, is also described as an intermediate in the biosynthesis of the “longevity vitamin” ergothioneine which possesses a sulfur at position C-2 of the imidazole [28]. Here again, the mechanism was shown to be radical. Radical-mediated enzymatic methylation has been well studied and involves radical SAM methyltransferases (RSMTs) of three different classes [29]. Interestingly, class B methyltransferases use cobalamin as a conduit of the methyl group to the substrate [30]. From the sponge, we were able to isolate good amounts of cobalamin and cyanocobalamin which is in accordance with the presence of this class of enzyme in the holobiont. As shown in Scheme 1, two molecules of *S*-Adenosyl Methionine would therefore be necessary to methylate the most stable radical at position C-2.

Compound **8** was assessed for cytotoxicity, using clonogenic assays, against HeLa (cervical cancer) cells and showed a significant bioactivity with a LC_50_ of 3.5 μM. 

## 3. Materials and Methods

### 3.1. General Experimental Procedures

Optical rotations were measured on a UNIPOL 1000 Polarimeter. UV and ECD measurements were obtained on a Chirascan (Applied Photophysics, Leatherhead, UK) spectrophotometer. NMR experiments were measured on a 600 MHz equipped with a cryoprobe (Varian) or 500 MHz (Agilent, Cheadle, UK). Chemical shifts (δ in ppm) are referenced to trace methanol (*δ*_H_ 3.34, *δ*_C_ 49.5) for NMR in D_2_O (>99.8 atom % D, Merck, Wicklow, Ireland) and residual proton and carbon signals (*δ*_H_ 3.31, *δ*_C_ 49.0) for NMRs in CD_3_OD (>99.8 atom % D, Merck). High Resolution Electrospray Ionization Mass Spectrometry (HRESIMS) data were obtained from a Q-ToF Agilent 6540 in ESI(+). Preparative HPLC was preformed using a PU-2087 Plus (Jasco, Dunmow, UK) equipped with a UV-Vis detector UV 2075 Plus and then by Agilent 1260 analytical HPLC series equipped with a DAD detector.

### 3.2. Biological Material

The specimen was collected from a depth of 809 m (48.6509° N, 10.4846° W) by the remotely operated vehicles Holland 1 during the CE16006 cruise of the RV Celtic Explorer. The sponge appeared as a white barrel sponge. In-situ pictures were taken to aid identification. All epibionts were removed and a small section was stored in 96% ethanol as a voucher specimen. The rest of the biomass was lyophilized and stored at −20 °C. 

### 3.3. Extraction and Isolation

The sponge (330 g) was ground using a ball mill and extracted with a mixture MeOH/CH_2_Cl_2_ (1:1) and ultrasonification. The crude extract (20.6 g) was fractioned using RP-C18 Vacuum Liquid Chromatography (VLC) into five fractions of decreasing polarity from F1(H_2_O), F2(H_2_O/MeOH (1:1)), F3 (H_2_O/MeOH (1:3)), F4 (MeOH) and F5 (MeOH/CH_2_Cl_2_ (1:1)). 

The water-methanol fraction (942.2 mg) was separated using RP-HPLC on a semi-preparative T3 column, 250 mm × 19 mm, 5 µm (Xselect, Waters, Milford, CT, USA), using a mobile phase of water (A) and methanol (B), both with 0.1% TFA and a flow rate of 5 mL/min. The gradient method was developed with a 33 min run time: isocratic at 8% B for 5 min, a liner gradient for 18 min to 65% B, isocratic at 65% for 5 min and returning to 8% B for 5 min. Compound 7 (RT 13.0 min, 2.87 mg) was collected at sufficient purity and compound **8** was collected as a mixture (52 mg). Repurification was carried out on a Waters 2695 HPLC using a semi-preparative reversed-phase amide column (Waters analytical BEH column 5 μm 10 mm × 250 mm) with 5 mL.min-1 flow rate and injections ranging from 10 μL to 60 μL with a gradient mobile phase of H_2_O (A) and CH_3_CN (B) both acidified with 0.1% *v*/*v* TFA. The gradient method was developed with a 19 min run time and gradient specifications: 2 min isocratic at 90% B, linear gradient for 13 min to 80% B, followed by an instant return to 90% B at which it was held for 4 min until completion. Compound 8 (RT 8.5 min; 2.10 mg) was obtained in enough purity for structural elucidation using NMR. 

Cyanocobalamin (**7**): Pink amorphous solid; UV (DAD) *λ*_max_ 360 nm and 270 nm; HRESIMS (+) *m*/*z* 1355.5719 [M + H]^+^ (calcd. For C_63_H_89_CoN_14_O_14_P, 1355.5752, Δ −2.1 ppm).

6-methylhercynine (**8**): White amorphous solid; [α]_20_^D^ +42°; UV (DAD) *λ*_max_ 254 nm; ^1^H-NMR and ^13^C NMR, see Table 1; HRESIMS (+) *m*/*z* 212.1394 [M + H]^+^ (calcd. For C_10_H_18_N_3_O_2_, 212.1394, Δ 0.0 ppm).

### 3.4. Computational Methods

A conformational analysis of **8** was performed using a Monte Carlo Minimum method (MCMM) and the molecular mechanics OPLS3 force field with an energy cut off of 5 kcal/mol in Schrodinger MacroModel [31]. These conformers were then optimized using DFT, at the M06-2X/6-31G(d,p) level in Gaussian 16, with the zero-point energy, electronic transition, and rational strength also calculated [32]. Following this, the ECD spectra for each conformer were calculated in Gaussian 16 at the B3LYP/6-311G(d,p) level. All DFT calculations were performed using a polarizable continuum solvation model [33]. The final ECD spectra were extracted, Boltzmann weighted and corrected by alignment with the UV spectra using the freely available software SpecDis 1.7 (version 1.71, SpecDis, Berlin, Germany) [34].

### 3.5. Molecular Network

LC-MS/MS data were acquired in Data dependent acquisition (DDA) mode on a HRESIMS- Q-ToF Agilent 6540 in ESI(+), using a C_18_ column (Xselect, Waters, Milford, CT, USA) with a mobile phase of water (A) and acetonitrile (B), both with 0.1% formic acid (FA) and a flow rate of 0.5 mL/min. The gradient method was developed with an 18 min run time: isocratic at 10% B for 2 min, a linear gradient for 10 min to 100% B, isocratic at 100% for 3 min and returning to 10% B over 1 min and remaining isocratic at 10% B for 2 min. Parameters, conditions and spectra, used in a LC-MS/MS data acquisition are available in Table S1. 

Molecular networks were first created using the Feature-Based Molecular Networking (FBMN) workflow (on the GNPS platform (https://gnps.ucsd.edu accessed on 21 December 2021). The raw mass spectrometry data were converted to mzXML using Proteowizard (version 3.18212). MS data were subsequently processed in MZmine2 using its MS peak detection, chromatogram builder, chromatogram deconvolution, isotopic grouping, and feature alignment tools. The resulting spectra were manually validated to ensure all spectra were processed correctly. The feature quantification table and MS/MS spectral summary were exported to GNPS feature based workflow for analysis. 

Data filtering was carried out by removing MS/MS fragment ions within +/−17 Da of the precursor *m*/*z*. MS/MS spectra were window filtered by choosing only the top 6 fragment ions in the +/−50 Da window throughout the spectrum. Mass tolerance for the precursor ion and the MS/MS fragments was set at 0.05 Da. The molecular networks were then created where edges appeared in the network if more than 6 peaks were matched and awarded a cosine of above 0.7. Furthermore, edges between two nodes were only kept if each of the nodes appeared in the others top 10 most similar nodes. The maximin size for a molecular family was set to 100 (the lowest scoring edges were removed from clusters until size of the molecular family fell below the set threshold). The uploaded spectra were compared against GNPS spectral libraries. GNPS libraries were filtered using the same conditions as the input data. Matches between input data and library spectra were shown if they had a score of above 0.7 and at least 6 matched peaks. After file conversion using Proteowizard (version 3.18212), a molecular network was generated using the GNPS platform. The molecular network was visualized via Cytoscape (version 3.9.1).

### 3.6. Biological Activities

#### Clonogenic Survival Assay

HeLa cells purchased from ATCC were seeded into 96-well plates at a concentration of 200 cells/mL in Dulbecco’s Modified Eagle’s Medium (DMEM) supplemented with 10% Bovine serum. Drugs were added to the medium 24 h after seeding at the indicated concentrations, and the cells were allowed to grow for 10 days. The cells were fixed with a 10% methanol, 10% Acetic acid solution (in water) for 15 min at room temperature and stained with 1% crystal violet (in methanol) for 5 min. Excess dye was removed with water and the plates were allowed to dry at RT overnight. Cells were de-stained with Sorenson’s buffer (0.1 M sodium citrate, 50% ethanol). The colorimetric intensity of each solution was quantified using Gen5 software on a Synergy 2 (BioTek, Winooksi, VT, USA) plate reader (OD at 595 nm). 

## 4. Conclusions

Molecular networking proved to be a useful tool to explore the chemodiversity of the deep-sea tetractinellid sponge *Characella pachastrelloides*. First, the molecular network allowed us to discern characellide analogs present in too small quantities, and highly light sensitive poecillastrins. The discovery of a uniquely methylated derivative of hercynine, which proved active against a HeLa cell line, alongside cyanocobalamin, the most common synthetic form of vitamin B12, raised interesting questions about its biosynthetic origin. The chemodiversity of *Characella pachastrelloides* demonstrates the potential of deep-sea sponges, particularly tetractinellids, in biodiscovery. It is likely that some of the compounds, for example the cyanocobalamin, were produced by the sponge microbiome. Given the difficulties of culturing deep-sea microbes, collecting macrofauna can be an effective method of sampling deep-sea microbial diversity.

## Figures and Tables

**Table 1:** NMR spectroscopic data for compound **8** in D_2_O (500 MHz for ^1^H-NMR data and 125 MHz for ^13^C-NMR).

No.	6-Methylhercynine (8)	
	*δ_H_*, Mult. (J in Hz)	*δ*_C_
1	-	169.5
2	3.98, dd (12.0, 3.5)	76.5
3	3.46, dd (14.0, 3.5)	22.6
	3.36, dd (14.0, 12.0)	
4	-	127.7
6	-	144.0
8	7.29, s	118.8
9	3.32, s	52.2
10	2.73, s	15.3

## Data Availability

Feature-based molecular network: https://gnps.ucsd.edu/ProteoSAFe/status.jsp?task=18fcc78f96fd40f189b99a46fedf77b3 accessed on 21 December 2021. Classical molecular network: https://gnps.ucsd.edu/ProteoSAFe/status.jsp?task=5785a70c1741419793a5898079ebf64d accessed on 21 December 2021.

## Supplementary Materials

The following are available online at https://www.mdpi.com/article/10.3390/md20010052/s1, Table S1: Conditions and parameters used from HRESIMS analysis on *Characella pachastrelloides* using a UPLC-Q-ToF system for DDA LC-MSMS analysis. Figures S1–S17: HRESIMS NMR and UV spectra of **5**, **7** and **8**.
