# Supplementary material for: Unveiling the Chemical Diversity of the Deep-Sea Sponge Characella pachastrelloides
Source: Mar Drugs. 2022 Jan 5;20(1):52. doi: 10.3390/md20010052 (PMC8779493; doi:10.3390/md20010052)
Supplement: Supplementary file 1 [file marinedrugs-20-00052-s001.zip › marinedrugs-1522185-supplementary.pdf]

Supplementary information

Unveiling the chemical diversity of the deep-sea sponge *Characella pachastrelloides*

Sam Afoullouss [1,2], Anthony R. Sanchez [3], Laurence K. Jennings[1], Younghoon Kee[3], A. Louise Allcock[2], and Olivier P. Thomas [1,*]

[1] Marine Biodiscovery, School of Chemistry and Ryan Institute, National University of Ireland Galway (NUI Galway), University Road, H91TK33 Galway, Ireland·
[2] School of Natural Sciences and Ryan Institute, National University of Ireland Galway (NUI Galway), University Road, H91TK33 Galway, Ireland
[3] Department of Cell Biology, Microbiology, and Molecular Biology, University of South Florida, 4202 E.Fowler Ave., ISA2015, Tampa, FL 33620, USA; anthony.sanchez@austin.utexas.edu (A.R.S.); ykee@dgist.ac.kr (Y.K.)

Table S1: Conditions and parameters used from HRESIMS analysis on *Characella pachastrelloides* using a UPLC-Q-ToF system for DDA LC-MSMS analysis......3

Figure S1: Feature based molecular network with a zoom in on characellide cluster. Two known compounds characellide A (**1**) and characellide C(**3**) were annotated in the network. Characellides 854 and 868 are predicated to be present by examination of changes in fragments masses. MS/MS spectra of the characellides are displayed highlighting the key fragments. Fragmentation patterns of the characellides show the structures of the corresponding fragments......4
Figure S2: 1H-NMR spectra of mixture containing poecillastrin H (**5**) in CD_3OD (500 MHz)......5
Figure S3 : UPLC-UV (370 nm) chromatogram of the subfraction SA-1826-F3-P6 containing poecillastrin E & H (**5**,**6**)......5
Figure S4: HRESIMS spectra of poecillastrin H (**5**)......6
Figure S5: HRESIMS spectra of poecillastrin E (**6**)......6
Figure S6: ^{1}H-NMR spectra of cyanocobalamin standard (top) and isolated cyanocobalamin (**7**) (bottom) in CD_3OD (600 MHz)......7
Figure S7: HSQC spectra of cyanocobalamin standard (top) and isolated cyanocobalamin (**7**) (bottom) in CD_3OD (600 MHz)......8
Figure S8: HRESIMS spectra of cyanocobalamin (**7**)......8
Figure S9: ^{1}H-NMR spectra of 6-methyl hercynine (**8**) in D_2O (500 MHz)......9
Figure S10: ^{1}H-^{1}H COSY NMR spectra of 6-methyl hercynine (**8**) in D_2O (500 MHz)......9
Figure S11: ^{13}C NMR spectra of 6-methyl hercynine (**8**) in D_2O (125 MHz)......10
Figure S12: ^{1}H-^{13}C HSQC NMR spectra of 6-methyl hercynine (**8**) in D_2O (500 MHz)......10
Figure S13: HMBC NMR spectra of 6-methyl hercynine (**8**) in D_2O (500 MHz)......11
Figure S14: HRESIMS spectra of 6-methyl hercynine (**8**)......11
Figure S 15: Experimental (solid black) and calculated ECD spectra of compound (**8**) for enantiomer 2S (dashed red) and 2R (dashed blue); and zwitterion (left), monocharged (centre), and double charged (right)......11
Figure S16: Experimental (solid black) and calculated UV spectra of compound (**8**) for enantiomer 2S (dashed red) and 2R (dashed blue); and zwitterion (left), monocharged (centre), and double charged (right)......12

Figure S17: HeLa cells were treated with 6-methylhercynine (red) or DMSO (black) at the indicated concentrations for 10 days. Cell survival was measured by crystal violet staining. Error bars represent 95% confidence interval from 3 biological replicate: 12

Parameter	Condition
**Sample Concentration**	2 mg/ mL
**Liquid Chromatography Duration**	18 min
**Collision Energy**	30 eV
**Precursor Per Cycle**	3
**Gas Temperature**	300 °C
**Nebuliser Pressure**	30 PSI
**Sheath Gas Temperature**	310 °C
**Capillary Voltage**	3000 V
**Nozzle Voltage**	750 V
**Fragmentor Voltage**	140 V
**Skimmer Voltage**	55 V

Table S1: Conditions and parameters used from HRESIMS analysis on *Characella pachastrelloides* using a UPLC-Q-ToF system for DDA LC-MSMS analysis.

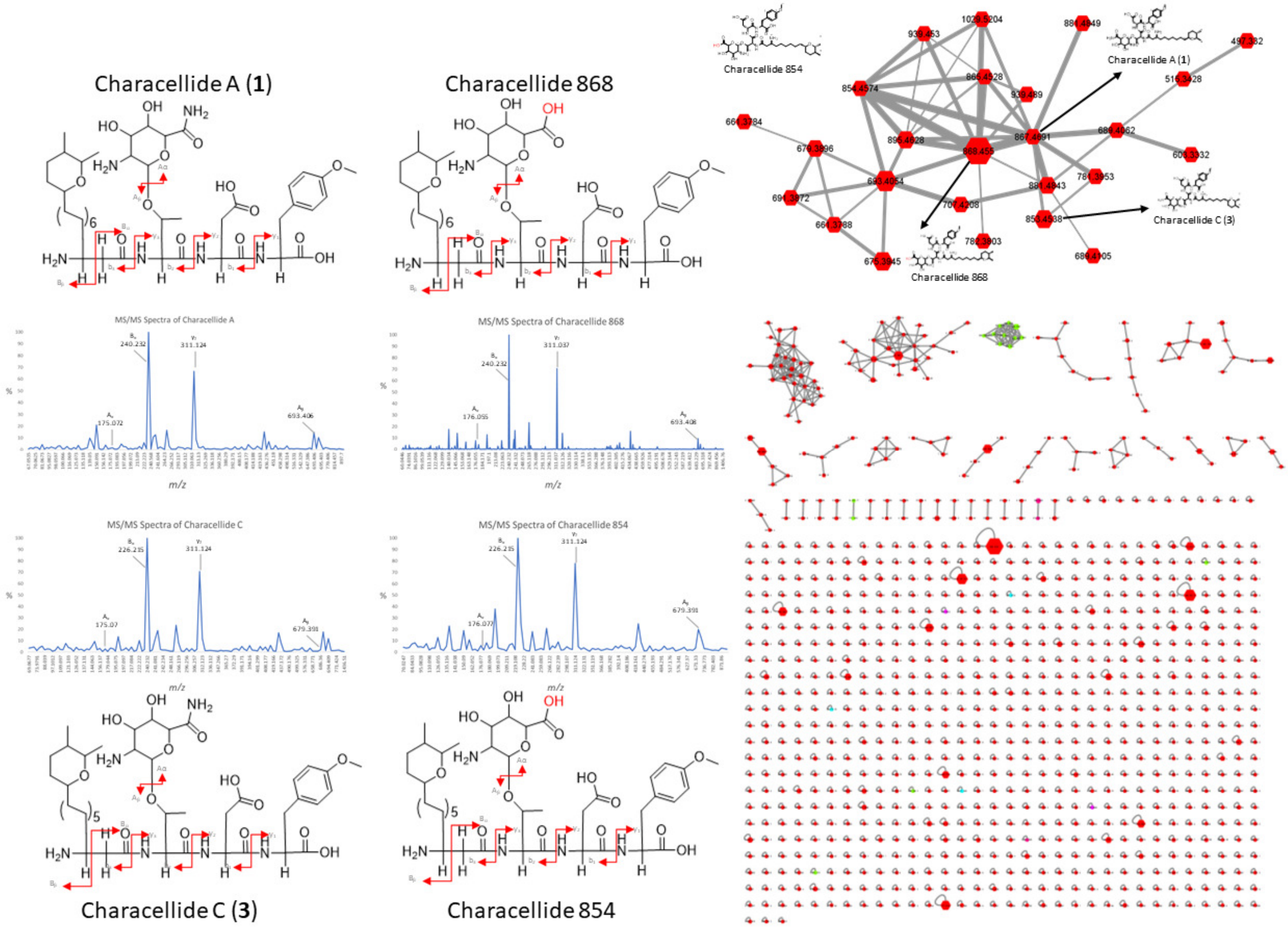

Figure S1: Feature based molecular network with a zoom in on characellide cluster. Two known compounds characellide A (**1**) and characellide C(**3**) were annotated in the network. Characellides 854 and 868 are predicated to be present by examination of changes in fragments masses. MS/MS spectra of the characellides are displayed highlighting the key fragments. Fragmentation patterns of the characellides show the structures of the corresponding fragments.

Poecillastrins 5 and 6

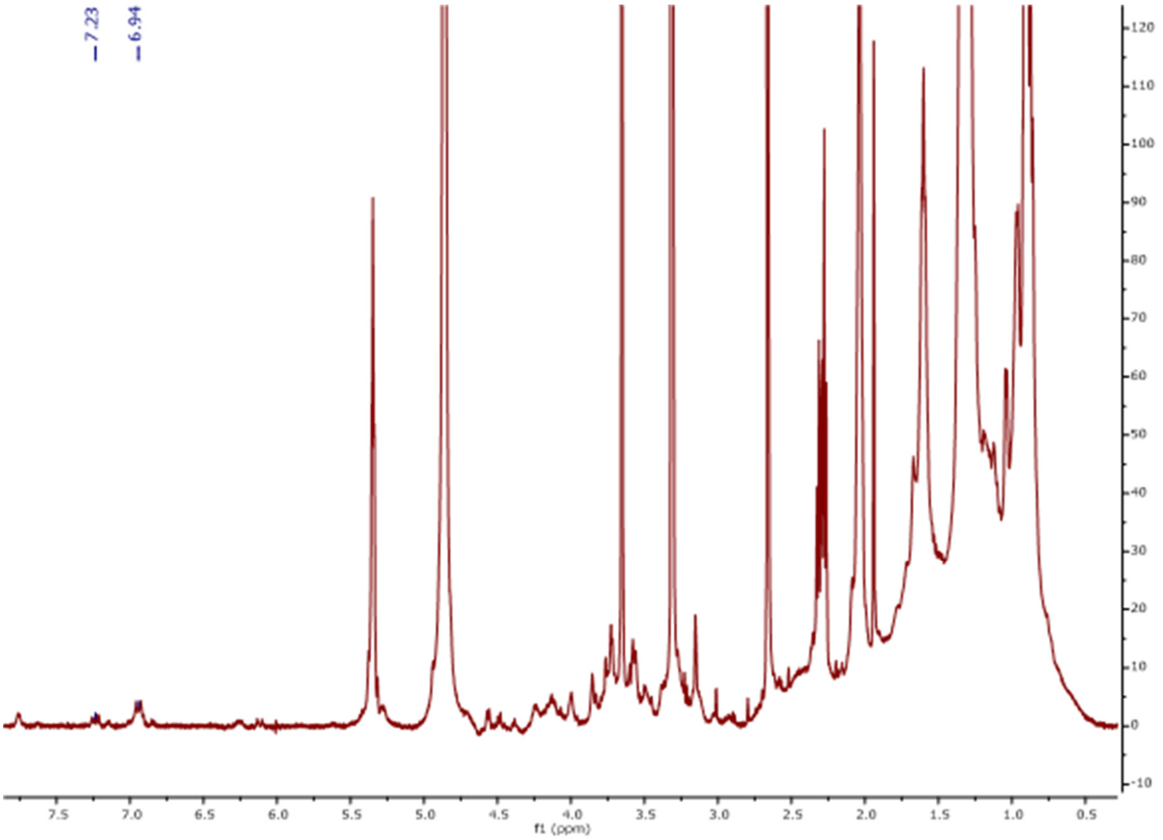

Figure S2: 1H-NMR spectra of mixture containing poecillastrin H (**5**) in CD_3OD (500 MHz)

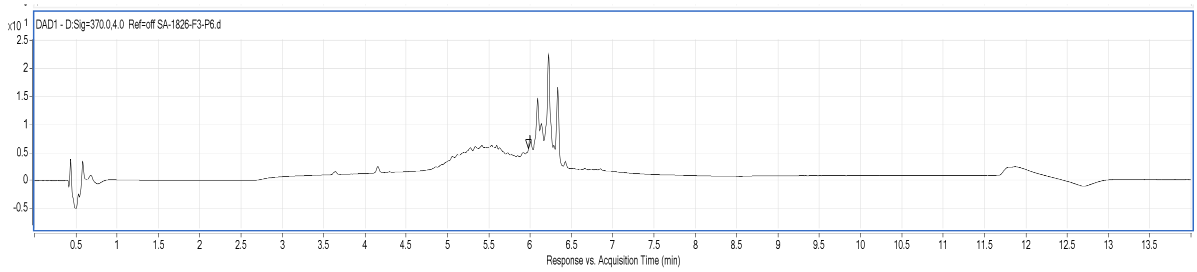

Figure S3 : UPLC-UV (370 nm) chromatogram of the subfraction SA-1826-F3-P6 containing poecillastrin E & H (**5**,**6**).

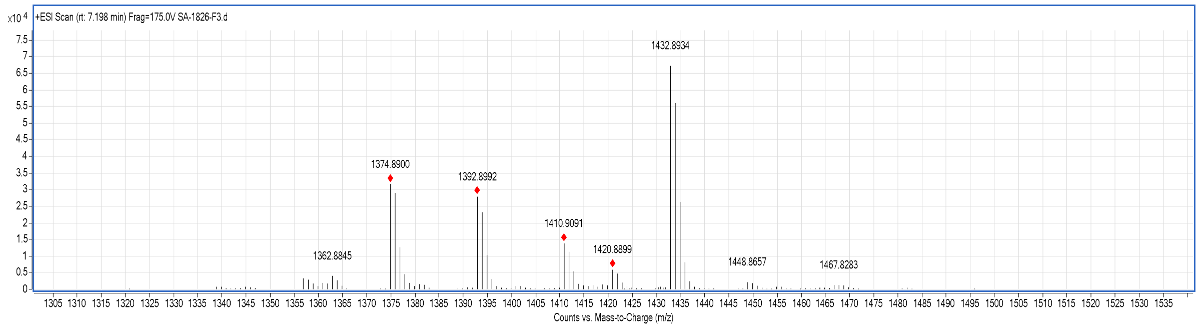

Figure S4: HRESIMS spectra of poecillastrin H (**5**).

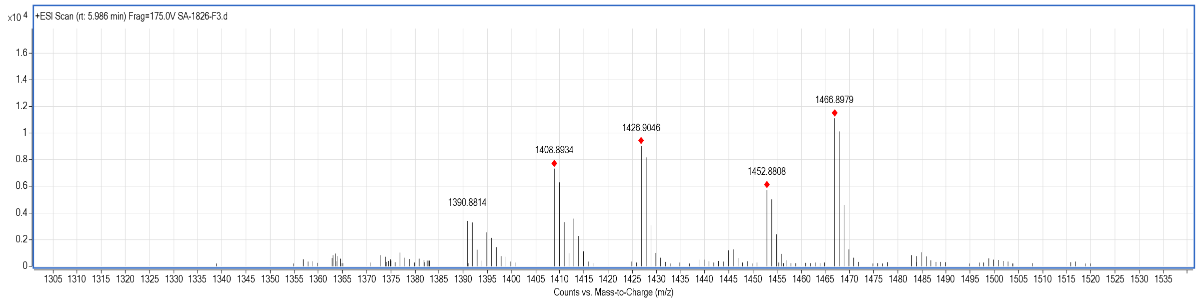

Figure S5: HRESIMS spectra of poecillastrin E (**6**).

Cyanocobalamin (7)

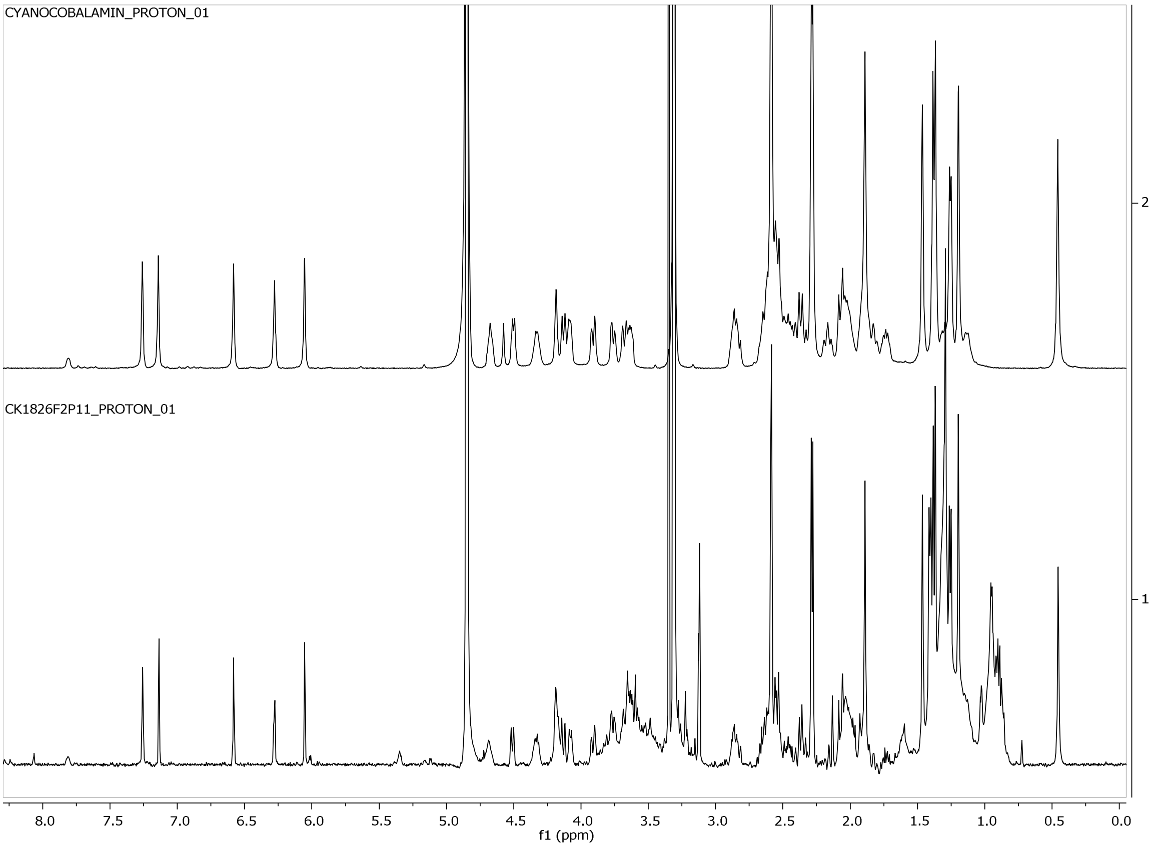

Figure S6: ^{1}H-NMR spectra of cyanocobalamin standard (top) and isolated cyanocobalamin (**7**) (bottom) in CD_3OD (600 MHz).

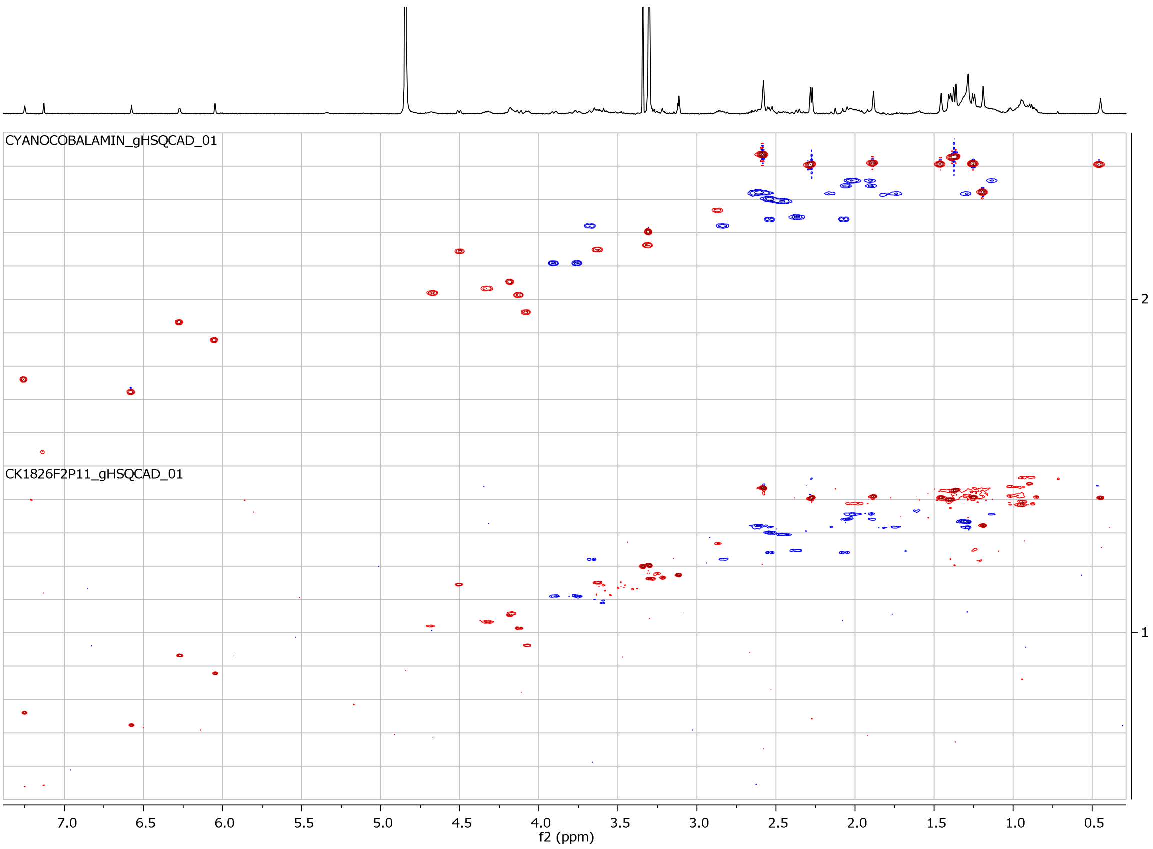

Figure S7: HSQC spectra of cyanocobalamin standard (top) and isolated cyanocobalamin (**7**) (bottom) in CD_3OD (600 MHz).

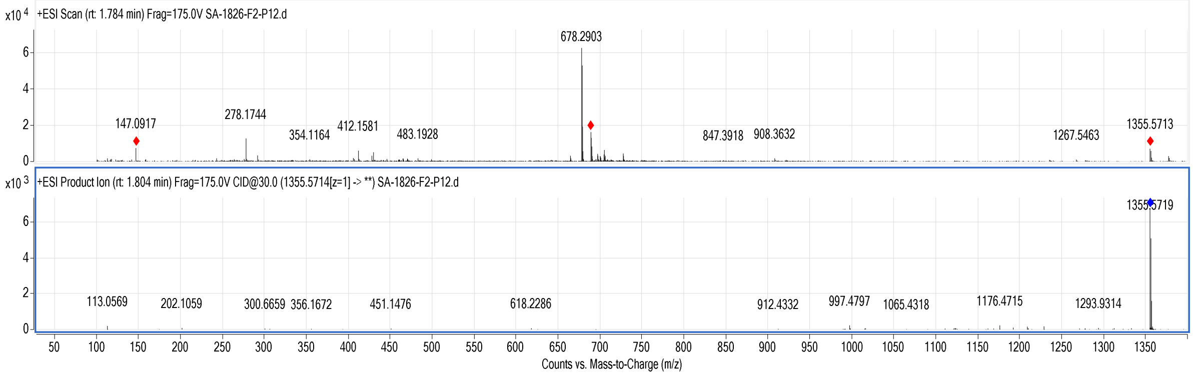

Figure S8: HRESIMS spectra of cyanocobalamin (**7**).

Betaine: 6-methylhercynine (8)

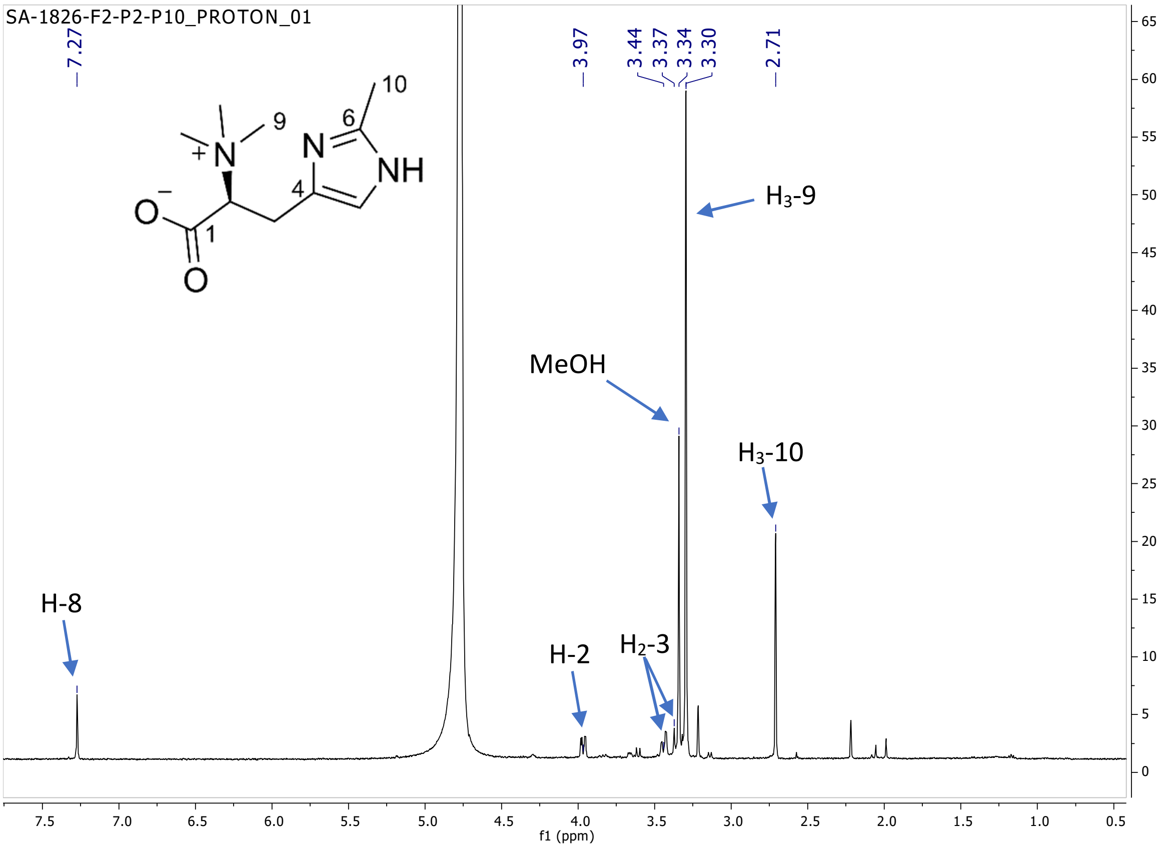

Figure S9: ^{1}H-NMR spectra of 6-methyl hercynine (**8**) in D_2O (500 MHz).

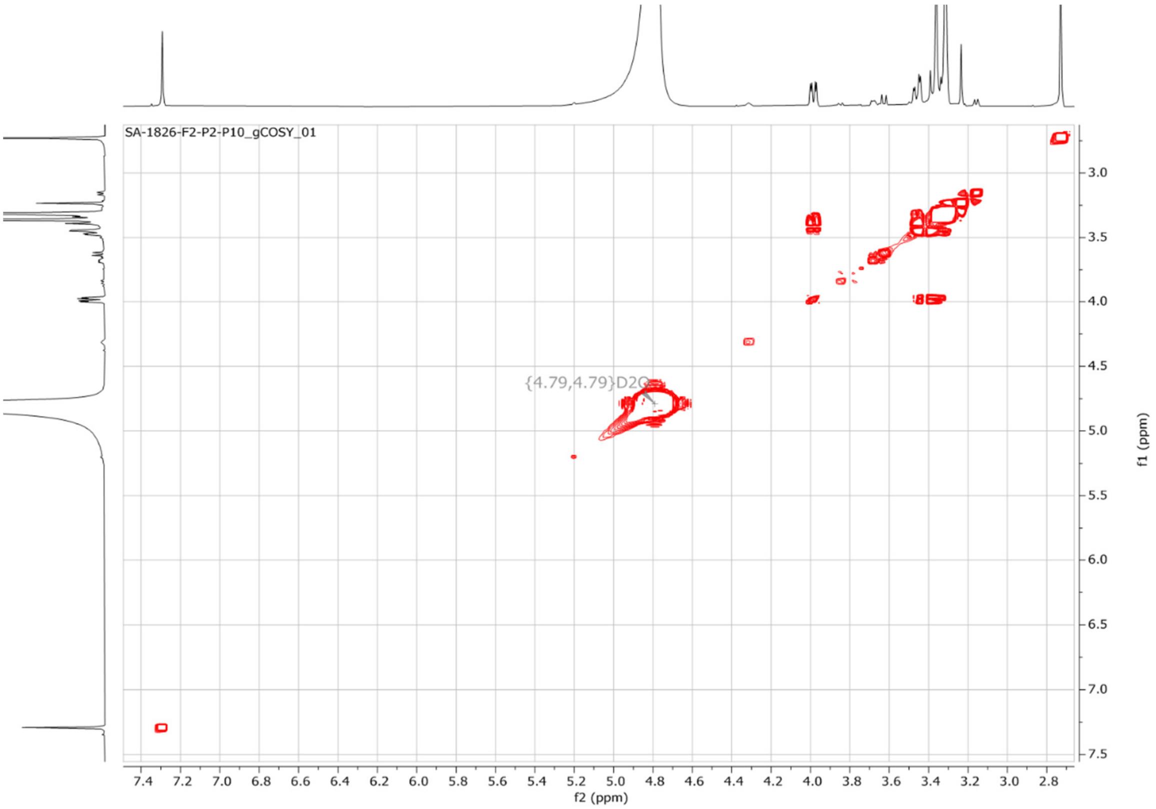

Figure S10: ^{1}H-^{1}H COSY NMR spectra of 6-methyl hercynine (**8**) in D_2O (500 MHz).

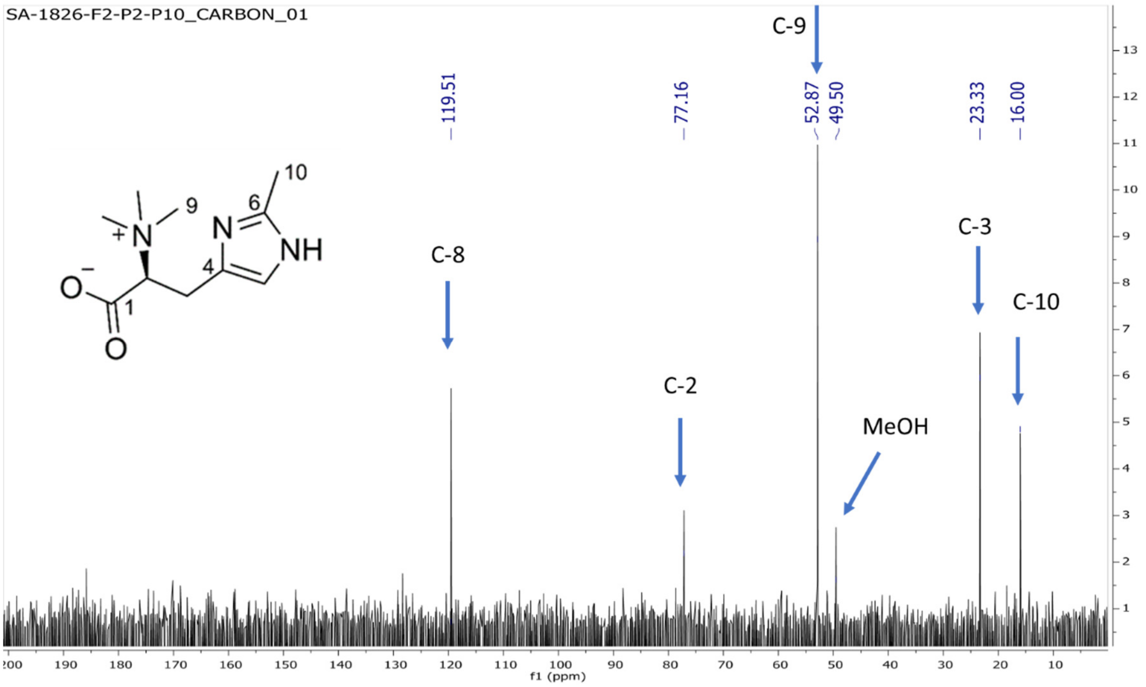

Figure S11: ^{13}C NMR spectra of 6-methyl hercynine (**8**) in D_2O (125 MHz).

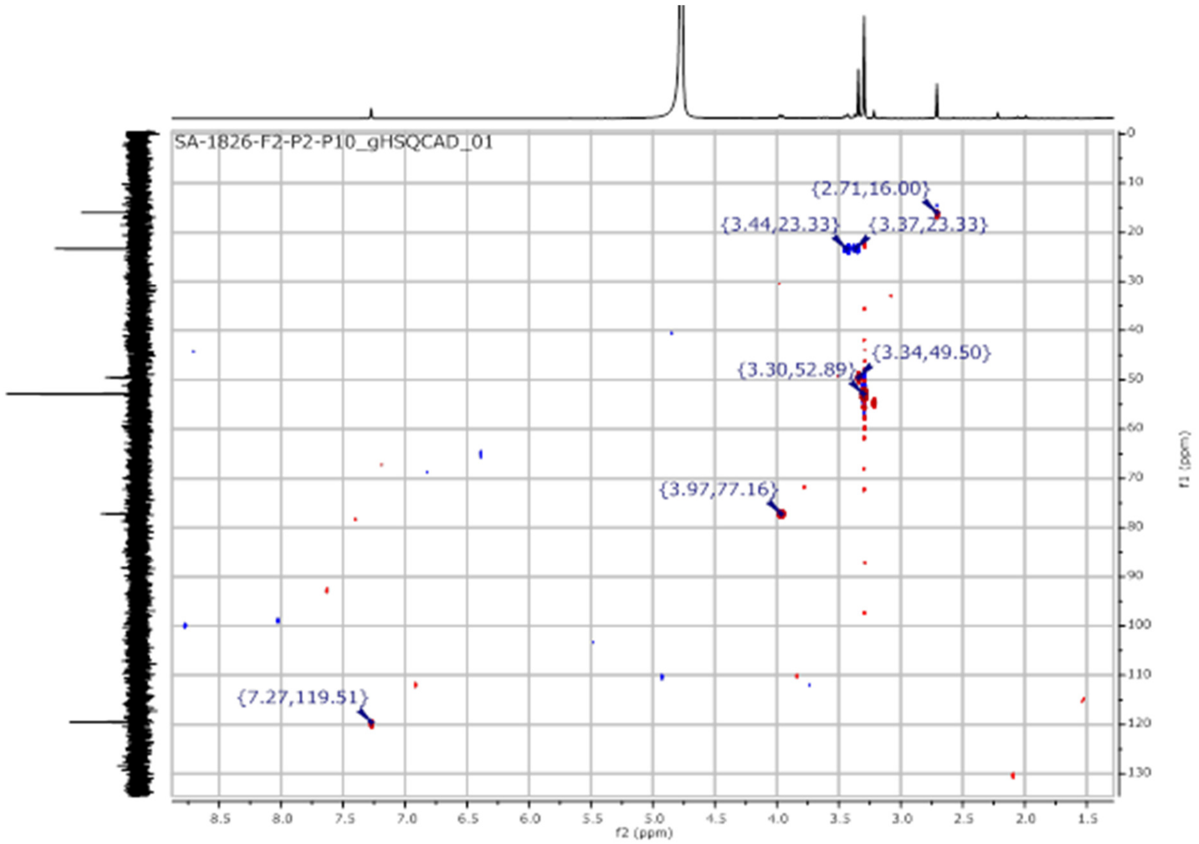

Figure S12: ^{1}H-^{13}C HSQC NMR spectra of 6-methyl hercynine (**8**) in D_2O (500 MHz).

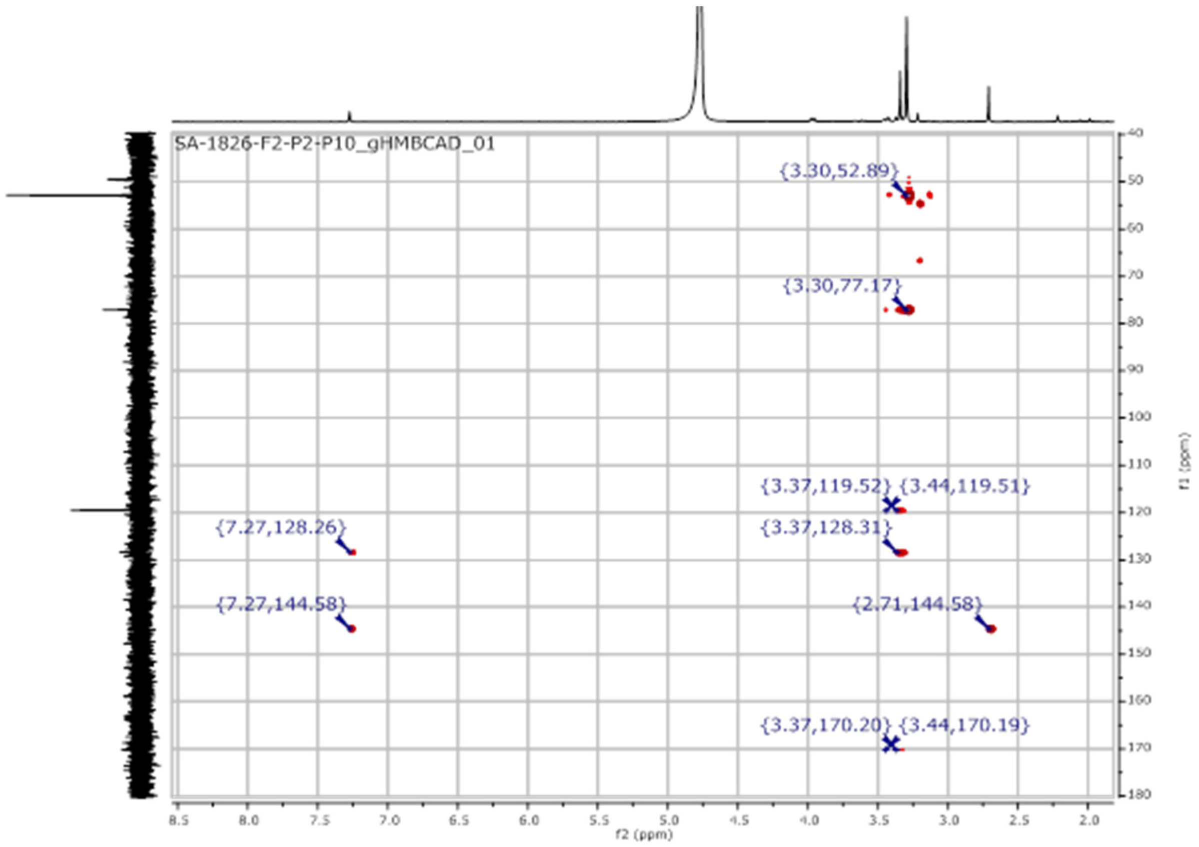

Figure S13: HMBC NMR spectra of 6-methyl hercynine (**8**) in D_2O (500 MHz).

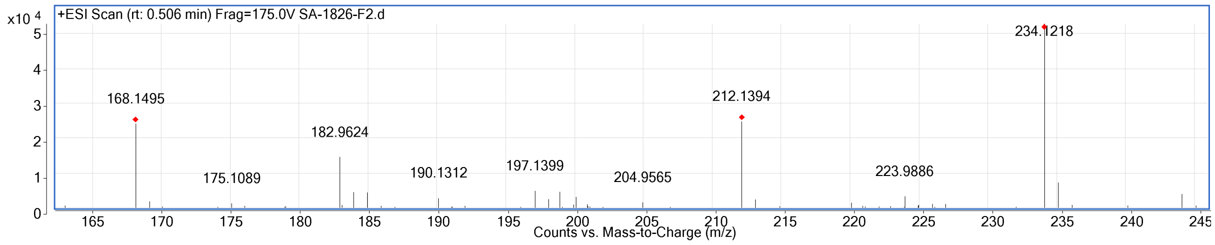

Figure S14: HRESIMS spectra of 6-methyl hercynine (**8**).

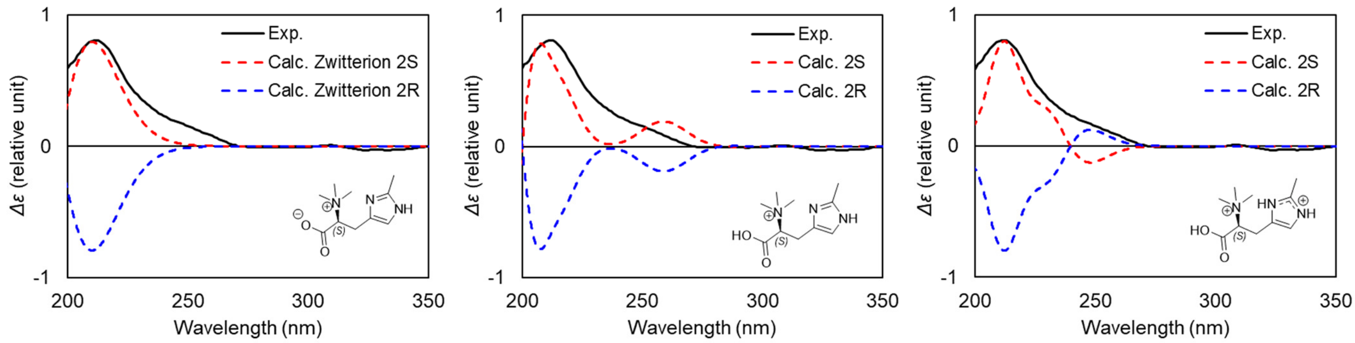

Figure S 15: Experimental (solid black) and calculated ECD spectra of compound (**8**) for enantiomer 2S (dashed red) and 2R (dashed blue); and zwitterion (left), monocharged (centre), and double charged (right).

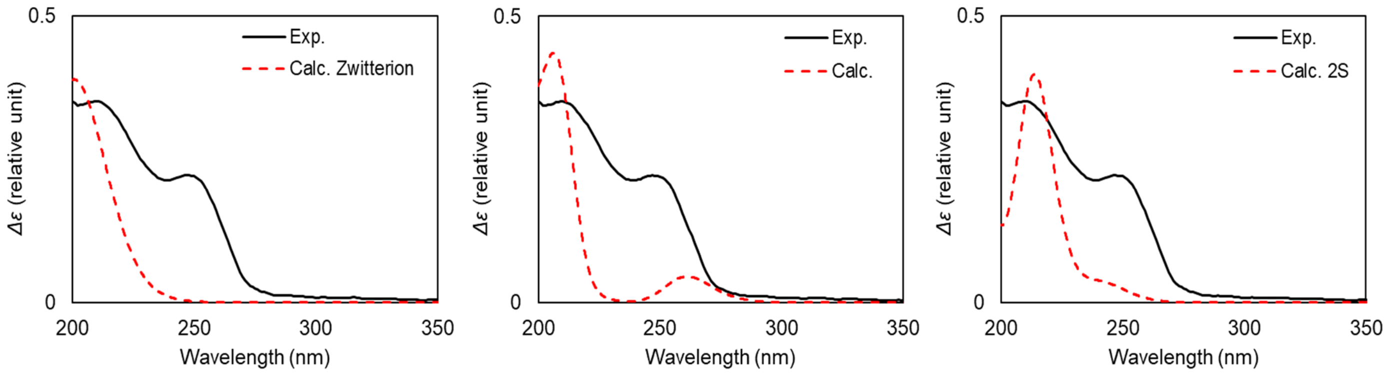

Figure S16: Experimental (solid black) and calculated UV spectra of compound (**8**) for enantiomer 2S (dashed red) and 2R (dashed blue); and zwitterion (left), monocharged (centre), and double charged (right).

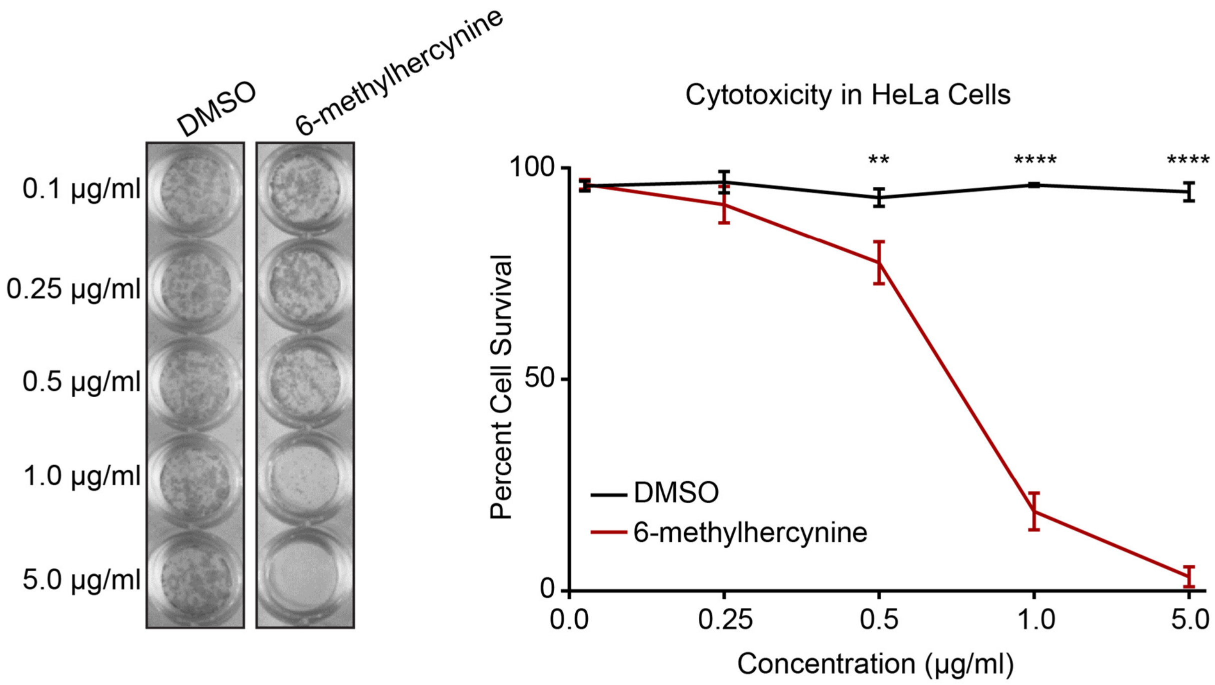

Figure S17: HeLa cells were treated with 6-methylhercynine (red) or DMSO (black) at the indicated concentrations for 10 days. Cell survival was measured by crystal violet staining. Error bars represent 95% confidence interval from 3 biological replicate:
